# Adherence to Trust Section 17 Leave Policy at High Dependency Unit and an Open Rehab Unit

**DOI:** 10.1192/bjo.2025.10665

**Published:** 2025-06-20

**Authors:** Heba Salem, Nidhi Gupta

**Affiliations:** BSMHFT, Birmingham, United Kingdom

## Abstract

**Aims:** Following initial audit on trust policy for section 17 leave in 2023, this second cycle aimed to reassess staff adherence with the trust section 17 leave policy as well as monitor the change in practice. The first cycle of audit was presented at the clinical forum, and all the professionals agreed about documentation of reflections on leave and urine drug screen (UDS) in electronic notes.

**Methods:** Data was collected retrospectively from electronic case notes/patient portal in July 2024, for all detained patients on both units. Fifteen patients were included in this audit. Section 17 leave was a Multidisciplinary team (MDT) decision.

**Results:** For all patients, a clear discussion regarding appropriateness of leave including current presentation and risk were documented. A clear physical description and photographs were completed for all the patients. The patients involved in the audit had utilized a total of 197 leaves in a one-month period. This included 33 escorted, 155 unescorted, 9 accompanied leaves. For accompanied leave, the accompanying person, and the purpose of the leave was recorded. For all leaves, restrictions and contingency plan were clearly documented on the leave prescription.

At the end of any leave period, staff reflection on the leave period were documented in 7.6% as compared with 20.14% in the first cycle of the audit. Staff scored 100% in recording patient feeling after the leave on patient portal. Out of 155 unescorted leaves, 92 leaves had search restriction. 100% searches were conducted which was an improvement from 95% in first cycle. UDS required as per leave prescription for 62 of unescorted leaves were completed and documented in 53.20%, which was an improvement from 17.20% in the first cycle.

**Conclusion:** The audit results show effective completion of risk assessment, physical description and making leave decisions as an MDT. There had been slight increase in the post leave search and UDS in comparison to the first cycle of audit (2023). However, staff reflection on electronic notes and UDS compliance still needs improvement.

Recommendations include increased training of staff in documentation, regular audits, and encouraging post leave evaluations to enhance the safety of section 17 leave process.

